# *De novo* assembly and annotation of three *Leptosphaeria* genomes using Oxford Nanopore MinION sequencing

**DOI:** 10.1038/sdata.2018.235

**Published:** 2018-11-06

**Authors:** Fabien Dutreux, Corinne Da Silva, Léo d’Agata, Arnaud Couloux, Elise J. Gay, Benjamin Istace, Nicolas Lapalu, Arnaud Lemainque, Juliette Linglin, Benjamin Noel, Patrick Wincker, Corinne Cruaud, Thierry Rouxel, Marie-Hélène Balesdent, Jean-Marc Aury

**Affiliations:** 1 Genoscope, Institut de Biologie François-Jacob, Commissariat à lʼEnergie Atomique (CEA), Université Paris-Saclay, F-91057 Evry, France; 2 UMR BIOGER, INRA, AgroParisTech, Université Paris-Saclay, Avenue Lucien Brétignières, BP 01, F-78850 Thiverval-Grignon, France; 3 Génomique Métabolique, Genoscope, Institut de Biologie François Jacob, Commissarait à lʼEnergie Atomique (CEA), CNRS, Université dʼEvry, Université Paris-Saclay, 91057 Evry, France

**Keywords:** Comparative genomics, DNA sequencing, Genome assembly algorithms

## Abstract

*Leptosphaeria maculans* and *Leptosphaeria biglobosa* are ascomycete phytopathogens of *Brassica napus* (oilseed rape, canola). Here we report the complete sequence of three Leptosphaeria genomes (*L. maculans* JN3, *L. maculans* Nz-T4 and *L. biglobosa* G12-14). Nz-T4 and G12-14 genome assemblies were generated *de novo* and the reference JN3 genome assembly was improved using Oxford Nanopore MinION reads. The new assembly of *L. biglobosa* showed the existence of AT rich regions and pointed to a genome compartmentalization previously unsuspected following Illumina sequencing. Moreover nanopore sequencing allowed us to generate a chromosome-level assembly for the *L. maculans* reference isolate, JN3. The genome annotation was supported by integrating conserved proteins and RNA sequencing from Leptosphaeria-infected samples. The newly produced high-quality assemblies and annotations of those three *Leptosphaeria* genomes will allow further studies, notably focused on the tripartite interaction between *L. maculans*, *L. biglobosa* and oilseed rape. The discovery of as yet unknown effectors will notably allow progress in *B. napus* breeding towards *L. maculans* resistance.

## Background & Summary

Fungi have an important ecological and economic role. Many of them are pathogens of animals or plants, especially *Leptosphaeria maculans* which is an ascomycete phytopathogen of *Brassica napus* (oilseed rape, canola). Two genomes of *Leptosphaeria* were already available, the first genome sequence of this fungus (JN3) was obtained in the 2000’s using Sanger sequencing^1^ and composed of 41 scaffolds totaling 43.76 Mb (excluding ambiguous bases). In-depth analysis of this genome showed an unusual compartmentalization between AT-rich and GC-equilibrated blocks termed isochores. The location of effector genes in the plastic, AT-rich compartments of the genome was instrumental in establishing the “two-speed fungal genome” paradigm^1^. According to this paradigm, phytopathogen genomes encompass a plastic compartment enriched in genes involved in niche adaptation. Similar genome organisation was later on found to be a commonality in numerous filamentous phytopathogens, but its identification was slowed down by the use of short-reads sequencing that tended to misrepresent the plastic, Transposable Element (TE)-rich genome in the assemblies. Thus the genome sequencing of *Leptosphaeria biglobosa* (B3.5), a species related to *L. maculans* resulted in a poor-quality assembly with complete absence of a putative dispensable genome^2^. This assembly of B3.5 was composed of 606 scaffolds totaling 29.44 Mb (excluding ambiguous bases).

**Figure 1:**
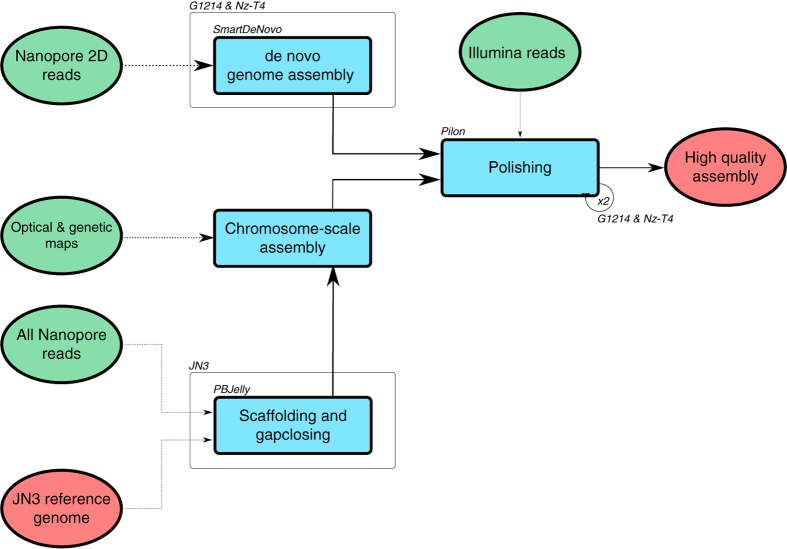
General description of the assembly workflow. Amelioration of the existing JN3 assembly and *de novo* assembly of Nz-T4 and G12-14 isolates.

**Figure 2:**
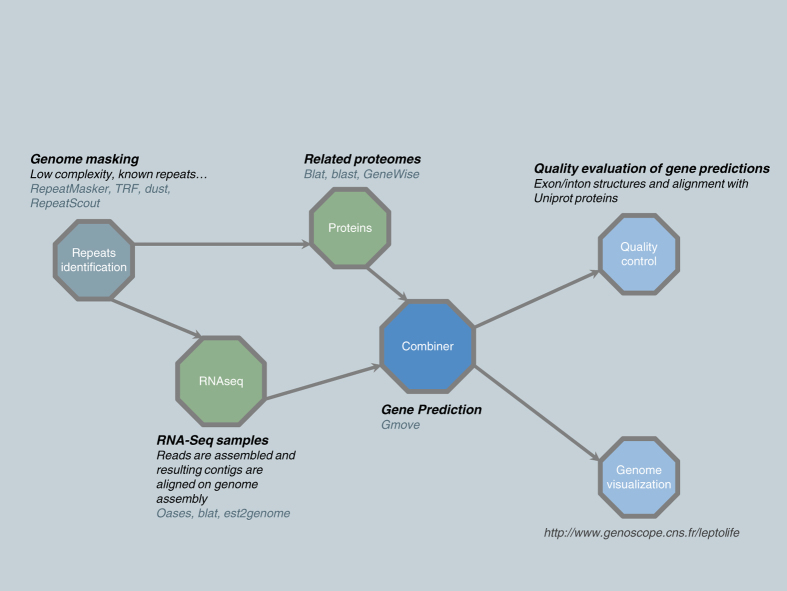
General description of the gene prediction workflow.

**Figure 3:**
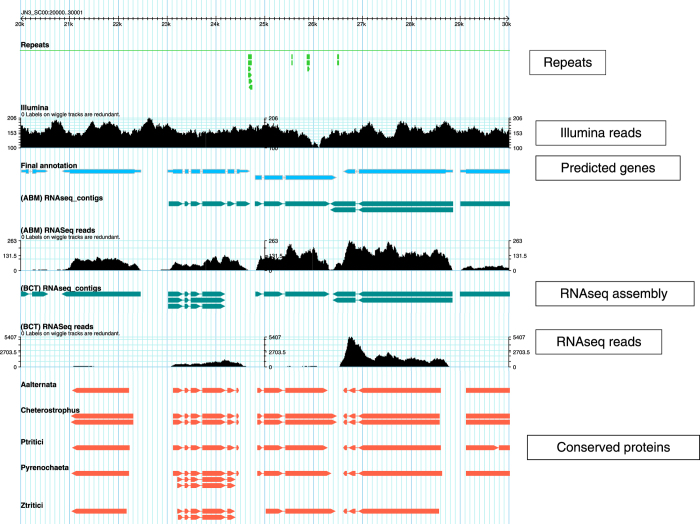
Genome browser database for the three *Leptosphaeria* isolates. The genome browser is available at http://www.genoscope.cns.fr/leptolife and contains repeats (green track), coverage of genomic reads (black wiggle), gene prediction (blue track), RNA contigs (dark green track) and protein homologies (salmon track).

Generally, when using short-read sequencing strategies, TE complicate the assembly step and it results in an under estimation of the repetitive content by collapsing similar copies of repetitions. In this study, we generated genome assembly and annotation of three *Leptosphaeria* isolates. To overcome the limitation of short reads, we generated long reads using the Oxford Nanopore technology (Data Citation 1, 2, 3). First, we resequenced the JN3 isolate with the objective of improving the existing assembly in terms of both contiguity and gaps closure (Fig. 1). And then we *de novo* sequenced and assembled two isolates *L. maculans* Nz-T4 and *L. biglobosa* G12-14 (Fig. 1). For each isolate, we generated long and short reads using respectively the MinION device from Oxford Nanopore and the Illumina sequencing technology (Tables 1, 2, 3). Reconciling manually the data from nanopore sequencing, optical and genetic maps allowed the generation of a chromosome-scale assembly for JN3 composed of 19 chromosomes plus the conditionally dispensable chromosome^3^, with only four missing telomeric extremities. The three genome assemblies were composed of 33, 288 and 156 scaffolds totaling 45.99 Mb, 43.42 Mb and 34.95 Mb for JN3, Nz-T4 and G12-14, respectively (Table 4). Furthermore, the gene prediction was supported by integrating conserved proteins and RNA sequencing from Leptosphaeria-infected samples (Fig. 2 and Data Citation 4). It resulted in 13,047, 14,026 and 12,678 predicted protein-coding genes for JN3, Nz-T4 and G12-14, respectively (Table 5 and Fig. 3).

## Methods

### Biological material

Two *L. maculans brassicae* isolates, JN3 (v23.1.3) and Nz-T4 were sequenced here. JN3 is the reference sequenced isolate^1^ of European origin. Nz-T4 has been used in previous genetic analyses^4^ and originates from New Zealand. The *L. biglobosa brassicae* isolate G12-14, also sequenced here, was isolated in 2014 in France (Thiverval-Grignon) as a single-ascospore isolate from naturally infected oilseed rape stubble. G12-14 was chosen as a new *L. biglobosa* reference isolate since the previously sequenced isolate, B3.5^2^, showed a very low level of aggressiveness when used in cotyledon inoculation tests. In contrast, G12-14 rapidly produced on oilseed rape plants typical and intense symptoms of *L. biglobosa brassicae*. In addition it was considered as a better representative of the current *L. biglobosa* populations infecting oilseed rape in France than B3.5.

For RNA sequencing, additional isolates were used, including *L. maculans* JN2 (v23.1.2), a sister isolate of JN3 with a high level of aggressiveness and the *L. biglobosa brassicae* reference isolate, B3.5^2^. Plant materials infected with *L. maculans* and/or *L. biglobosa* were obtained under various conditions (in the field or under controlled conditions) and with three different *B. napus* varieties. Naturally infected plant material was obtained in Thiverval-Grignon experimental fields as either infected stem base at the end of the growing season, sampled one week before harvest (variety Darmor-*Bzh*) or as whole stem residues sampled two weeks after harvest (variety Alpaga). Under controlled conditions, two varieties were used, Darmor-*Bzh* displaying a high level of field resistance to *L. maculans*, and Bristol, with a high level of susceptibility. Cotyledons and stems were inoculated as previously described^5^ and inoculated material was sampled at different time points after inoculation. For petiole inoculations, the petiole was cut under the leaf blade. Ten μL of inoculum (10^7^ pycnidiospores mL^−1^) were placed over the wound. Inoculated plants were incubated in darkness for 48 h at room temperature under saturated humidity, and then transferred to a growth chamber at 16 °C (night) and 24 °C (day), with a 16 h photoperiod. Infected petioles were sampled after 7 and 14 days post infection (dpi).

### RNA/DNA extraction

Fresh infected cotyledons or freeze-dried cultures of fungal mycelia were ground using a Retsch MM300 mixer mill in Eppendorf tubes, with one tungsten carbide bead per tube, for 45 s with 30 oscillations per second. Petioles were ground with a pestle and mortar. Stem base and stem residues were ground with a Retsch MM300 mixer mill using Zirconium oxide jars and beads, for 40 s with 30 oscillations per second. For RNA extraction, all grinding material was frozen with liquid nitrogen prior grinding. RNA extraction was then performed using the Trizol reagent (Invitrogen, Cergy Pontoise, France) as previously described^6^. DNA was extracted from freeze-dried material using the plant DNeasy mini-kit or the Wizard® Genomic DNA Purification Kit (Promega, Madison, WI, USA) according to the manufacturers’ instructions. Input DNA for the nanopore sequencing, based on long DNA molecules, was extracted from agarose plugs of concentrated pycnidiospores (>1.10^9^ spore ml^−1^) partially digested^1,3^. Briefly, fungal pycnidiospores were collected from V8-agar plates^3^ with sterile distilled water, centrifuged (15 min at 10000×g) and adjusted to 6.10^9^ spore mL^1^. The spore suspension was mixed with an equal volume of 2.5% low melting agarose (Seaplaque GTG® in TSE; Tris 25 mM, pH 7.5; Sorbitol 1 M, EDTA 25 mM) maintained at 40 °C. This suspension was poured in disposable plug molds (BioRad). After cooling, the resulting plugs were treated for 20 h in 0.5 M EDTA, SDS 10%, 1 mg.mL^−1^ proteinase K (Sigma) at 50 °C under mild stirring, then rinsed three times with 0.5 M EDTA, 50 °C and stored at 4 °C in 0.5 M EDTA. For a part of Nanopore sequencing runs, DNA was also extracted from freeze-dried mycelium ground in liquid nitrogen: cells have been then lysed using CTAB (Cetyltrimethylammonium bromide) with 2% of PVP (Polyvinylpyrrolidone) and 0.1% of 2-mercaptoéthanol and DNA was purified following a phenol-chloroform protocol.

### Illumina PCR-Free library preparation and sequencing

DNA (4.5 to 6 μg) was sonicated to a 100- to 1500-bp size range using a Covaris E210 sonicator (Covaris, Woburn, MA, USA). Fragments were end-repaired using the NEBNext End Repair Module (New England Biolabs, Ipswich, MA, USA) and 3΄-adenylated with the NEBNext dA-Tailing Module (New England Biolabs). Illumina adapters were then added using the NEBNext Quick Ligation Module (New England Biolabs) and ligation products were purified with AMPure XP beads (Beckmann Coulter Genomics, Danvers, MA, USA). Libraries were sequenced on an Illumina MiSeq (G12-14 and NzT4 genomes) or a HiSeq 2500 (JN3 genome) instrument (San Diego, CA, USA) using 250 base-length read chemistry in a paired-end mode.

### Illumina cDNA library preparation and sequencing

RNA-Seq library preparations were carried out from 1–2 μg total RNA using the TruSeq Stranded mRNA kit (Illumina, San Diego, CA, USA), which allows mRNA strand orientation (sequence reads occur in the same orientation as anti-sense RNA). Briefly, poly(A)^+^ RNA was selected with oligo(dT) beads, chemically fragmented and converted into single-stranded cDNA using random hexamer priming. Then, the second strand was generated to create double-stranded cDNA. cDNA were then 3′-adenylated, and Illumina adapters were added. Ligation products were PCR-amplified. Each library was sequenced using 101 bp paired end reads chemistry on a HiSeq2000 Illumina sequencer.

### Nanopore 8-kb and 20-kb libraries preparation

MinION sequencing libraries were prepared according to the nanopore Sequencing Kit protocol SQK-MAP006 or the Low Input Expansion Pack protocol for genomic DNA. Briefly, 100 ng to 1.5 μg of genomic DNA was sheared to approximately 8 Kb with g-TUBE (Covaris, Woburn, MA, USA) and cleaned-up using AMPure XP beads (Beckmann Coulter Genomics). For some libraries, DNA fragments were repaired using the NEBNext FFPE Repair Mix (New England Biolabs). After end-reparation and 3′-adenylation with the NEBNext® Ultra^TM^ II End Repair/dA-Tailing Module (New England Biolabs), sequencing adapters provided by Oxford Nanopore Technologies (Oxford Nanopore Technologies Ltd, UK) were ligated using Blunt/TA Ligase Master Mix (New England Biolabs). Libraries were then enriched using Dynabeads MyOne C1 Streptavidin (ThermoFisher Scientific, Wilmington, DE, USA).

Nanopore 20-Kb libraries were prepared according to the same protocol, with the exception that 250 ng to 2.5 μg of genomic DNA was sheared to approximately 20-kb with g-TUBE (Covaris).

### MinION flow cell preparation and sample loading

Each library was mixed with the fuel mix and the running buffer according to the SQK-MAP006 or the Low Input Expansion Pack protocols. The sequencing mix was then added to the R7.3 flowcell for a 48-hour run. Several loading schedules were tested, but the main one used for the MAP006 libraries has been the following: a first loading with 8 μl of the library, then three reloading after 5, 24 and 29 h of run with respectively 4 μl, 8 μl and 4 μl of the DNA library. Regarding the low-input libraries, the main schedule used was a first loading with 10 μl of the DNA library and a reloading after 24 hours of run time with 10 μl of the DNA library.

### MinION sequencing and basecalling

Read event data generated by MinKNOW control software (version 0.50.2.15 to 0.51.3.40) were base-called using the Metrichor software (version 2.34.3 to 2.39.3). The data generated (pores metrics, sequencing, and base-calling data) by MinION software were stored and organized using a Hierarchical Data Format. Three types of reads were obtained: template, complement, and two-directions (2D). The template and complement reads correspond to sequencing of the two DNA strands. Metrichor combines template and complement reads to produce a consensus (2D) sequence^7^. FASTA reads were extracted from MinION Hierarchical Data Format files using poretools^8^. Pass and fail reads were both considered as 1D reads. This dataset is described in Table 1 and (Genomic datasets, Data Citation 5).

### Genetic map

A genetic map was built using 93 progeny of the JN2×Nz-T4 *in vitro* cross^9^. A total of 150 polymorphic micro- and minisatellite markers, three biological markers (Mat locus, *AvrLm3* and *AvrLm7*) and eight markers designed at the junction between two repeated elements^10^ were first used to generate an unsaturated genetic map using Mapmaker 3.0, with a LOD score of 3 and a maximum genetic distance of 20 cM. The 93 progeny isolates and the two parental isolates were also sequenced (2 × 100 bp illumina sequencing; 48 samples per line; Beckman Coulter Genomics). SNP calling between parental isolates was done as described in the study^11^ following mapping of the reads on the JN3 reference genome. A total of 20,103 reliable SNPs were detected and assigned to one of the two parental isolate in the progeny. These SNPs were converted into 2,104 Bins. From all these markers, a saturated map comprising 20 linkage groups was constructed using MSTmap online.

### Long reads-based de novo assembly of *L. biglobosa* G12-14 and *L. maculans* Nz-T4

R7.3 nanopore 2D reads (Table 3) were assembled into contigs using SmartDeNovo (https://github.com/ruanjue/smartdenovo), with a k-mer size parameter set to 21. Eventually, the consensus of the assembly was polished by aligning the Illumina paired-end 2 × 250 bp reads (Table 2) with BWA mem^12^ and using the resulting alignment file as input for Pilon^13^. We iteratively polished twice the assembly.

*L. biglobosa* G12-14 final assembly had a cumulative size of 34.9 Mb and a N50 equal to 462 kb. *L. maculans* Nz-T4 final assembly had a cumulative size of 43.4 Mb and a N50 equal to 383 kb. Further usual metrics regarding those assemblies are provided in Table 4.

### Genome assembly update of *L. maculans* JN3

With 33 scaffolds, a N50 equal to 2.40 Mb and a cumulative size of 44.8 Mb, the JN3 rearranged reference genome was already contiguous enough for our analyses. However, it contained gaps that represented 1.1 Mb in total. In addition, both the optical map^2^ and the high-density genetic map indicated that nine mis-assembled super contigs had to be either splitted or fused. The reference genome was scaffolded and gap-filled using PBJelly^14^ with the entire nanopore dataset as input (Table 3). As a result we obtained 33 scaffolds with a cumulative size of 45.8 Mb, the N50 increased to 2.43 Mb and we decreased by half the number of N’s (570 kb).

As the R7.3 nanopore reads were prone to errors, particularly in the homopolymeric regions^15^, we polished the consensus of the gap closed assembly by aligning 150X of Illumina paired-end 2 × 250 bp reads with BWA mem^12^ and giving the resulting alignment file to Pilon^13^. The final assembly had a cumulative size of 45.99 Mb with 1% of unknown bases (Table 4).

### Genome annotation

The gene prediction of the three *Leptosphaeria* genomes was done by combining gene models information computed from transcript and protein alignments (Fig. 2).

First, repeated sequences in the reference genomes were masked using multiple tools. Tandem repeats were identified and masked using Tandem Repeats Finder^16^. Low complexity regions were identified and masked using Dust^17^. Interspersed repeats and other low complexity sequences were masked using RepeatMasker (http://www.repeatmasker.org). Furthermore 121 known transposable elements (identified in the *L. maculans*-*L. biglobosa* species complex in a previous study^2^) were also masked using RepeatMasker.

The *Alternaria alternata*, *Cochliobolus heterostrophus*, *Pyrenochaeta* sp., *Pyrenophora tritici-repentis* and *Zymoseptoria tritici* proteomes included in UniProt (version available as of 07 September 2016) were used to detect conserved proteins in *L. maculans* JN3, *L. maculans* Nz-T4 and *L. biglobosa* G12-14. *L. maculans* JN3 and swissprot proteins (release available as of 30 November 2016) were also used as a resource to detect conserved proteins in *L. maculans* Nz-T4 and *L. biglobosa* G12-14.

Those proteins were first aligned to *L. maculans* JN3, *L. maculans* Nz-T4 and *L. biglobosa* G12-14 genomes assemblies using BLAT^18^. BLAT results were then filtered as follows: for each protein, the best match (based on the BLAT score) and other matches with a score > = 90% of the score of the best match were retained. Genewise^19^ was then used to refine matches and identify exon-intron boundaries and alignment were filtered out if less than 50% of the protein length was aligned.

RNAseq reads from 23 samples were assembled using Velvet^20^ 1.2.07 and Oases^21^ 0.2.08, using a k-mer size of 63 bp. The results of this assembly for each sample are summarized in (Transcriptomic datasets, Data Citation 5). Reads were mapped back to the contigs with BWA-mem^12^ and the consistent paired-end reads were selected. Chimeric contigs were identified and splitted (uncovered regions) based on coverage information from consistent paired-end reads. Moreover, open reading frames (ORF) and domains were searched using respectively TransDecoder^22^ and CDDsearch^23^. We only allowed breaks outside ORF and domains. At the end, the read strand information was used to correctly orient the RNA-Seq contigs. In a second step, RNAseq contigs were aligned to the relevant genome assembly (*L. maculans* JN3, *L. maculans* Nz-T4, *L. biglobosa* G12-14) using BLAT. The best matches (based on BLAT score) for each contig and other matches with a score >  = 90% of the score of the best match were selected. Then, Est2genome^24^ was used to refine the alignments and we kept alignments with an identity percent > = 95% and at least 80% of the RNA-Seq contig that can be aligned (Transcriptomic datasets, Data Citation 5).

Gene predictions from mRNA and proteomes were given as input to the GMOVE combiner^25^ to build the gene models. This tool considers biological data such as RNA-seq from the organisms of interest and proteome from homologous species. Proteins give information about the frame of the coding sequence (CDS) and RNA-seq helps to find splicing sites with more accurate alignments. This tool is designed for all eukaryotes and *de novo* studies with no need of calibration pre-processing. After gene models were produced by GMOVE, a final filter was applied to remove artefactual untranslated regions (UTRs). UTRs were trimmed if it overlapped CDS in predicted gene models for more than 10 bases. Moreover, UTRs overlapping other UTRs for more than 10 bases were also trimmed to avoid large and potentially false UTRs. Final metrics of the gene prediction are available in Table 5 and Fig. 3 is a screenshot of the genome browser.

### Code availability

All the tools used for this study are cited in the method section and they were used with default settings unless options were specified. The in-house tools to clean illumina data are available from the following website: http://www.genoscope.cns.fr/fastxtend/. The integration of resources in the gene prediction workflow was achieved using GMOVE: http://www.genoscope.cns.fr/gmove/.

## Data Records

The authors declare that all data reported herein are fully and freely available from the date of publication. Genomic data have been deposited in the ENA repository (Genomic datasets, Data Citation 5): *L. maculans* JN3 (Data Citation 1), *L. maculans* Nz-T4 (Data Citation 2) and *L. biglobosa* G12-14 (Data Citation 3). RNA data used for gene prediction have been deposited in the ENA repository (Data Citation 4 and Transcriptomic datasets, Data Citation 5). Moreover, a dedicated website brings together all the information: accession numbers, assembly and annotation files as well as a genome browser (Fig. 3): http://www.genoscope.cns.fr/leptolife.

## Technical Validation

### DNA and RNA sample quality

DNA quality was assessed using 1% agarose gel. RNA integrity was assessed using an Agilent 2100 Bioanalyzer electrophoresis system (Agilent, Santa Clara, CA, USA).

### Illumina libraries

Ready-to-sequence Illumina libraries were quantified by qPCR using the KAPA Library Quantification Kit for Illumina Libraries (KapaBiosystems, Wilmington, MA, USA), and libraries profiles evaluated with an Agilent 2100 Bioanalyzer (Agilent Technologies, Santa Clara, CA, USA).

### Illumina reads processing and quality filtering

After the Illumina sequencing, an in-house quality control process was applied to the reads that passed the Illumina quality filters. The first step discards low-quality nucleotides (Q < 20) from both ends of the reads. Next, Illumina sequencing adapters and primer sequences were removed from the reads. Then, reads shorter than 30 nucleotides after trimming were discarded. These trimming and removal steps were achieved using in-house-designed software based on the FastX package. The last step identifies and discards read pairs that mapped to the phage phiX genome, using SOAP^26^ and the phiX reference sequence (GenBank: NC_001422.1). This processing resulted in high-quality data and improvement of the subsequent analyses. This dataset is described in Table 2 and (Genomic datasets, Data Citation 5). A specific filter aiming to remove ribosomal reads was applied to data generated from RNA samples. The reads and their mates that mapped onto a ribosomal sequences database were filtered using SortMeRNA^27^. It contains different rRNA databases and we used it to split the data into two files: rRNA reads in a file (ribo_clean) and other reads in another file (noribo_clean). This dataset is described in (Transcriptomic datasets, Data Citation 5).

### Impact of the gap closing procedure on the JN3 assembly

The JN3 assembly was gap closed using PBJelly and we detected several duplicated regions of several Kb in this gap-closed assembly that were not present in the previous assembly. We first detected tandemly duplicated genes using Blast and revealed 28 candidate regions totaling 150 kb in length with a median size of 5.5 kb and covering 63 genes. Further investigations of these regions using genomic data coverage revealed that they were artefactual duplicates created by PBJelly. We mapped both Illumina and nanopore reads onto the gap-closed assembly and highlighted genomic regions without illumina and nanopore reads coverage. This method revealed 30 regions with a cumulative size of 185 kb and a median size of 5.1 kb spanning a total of 43 genes. Finally, after a careful expertise using the genome browser (Fig. 3), the genomic regions that presented duplicated genes and no proper read support were masked and overlapping genes were filtered out. In total, we masked 41 regions with a cumulative size of 264 kb and a median size of 5.5 kb that spanned 81 genes (Duplicated genomic regions, Data Citation 5).

### Contamination detection

In order to detect possible contamination in our assemblies, we first ran the metagene^28^ gene predictor on NzT4 and G12-14 contigs and on JN3 scaffolds. Then, we aligned all predicted proteins against the NR database using blastp. Hits were then filtered by their e-value to only conserve the best hit with an e-value of at least 10^−10^. Finally, a contig or scaffold was flagged as non-contaminant if at least 50% of its predicted proteins were assigned as fungi. Following this procedure, we couldn’t detect any contaminant sequences in JN3 and G12-14 assemblies. We detected 13 small contigs that were not assigned as fungi in the NzT4 assembly, but further investigations (blat against the JN3 assembly and blastx using the NCBI blast server) revealed that they were not contaminants.

### Comparison with existing assemblies

An AT/GC region segmentation was performed on all assemblies with OcculterCut^29^ to detect potential genome compartmentalization as described in the following study^1^. *L. maculans* JN3 and Nz-T4 isolates showed similar genome architecture with a third of their assembly dispatched into AT blocks of 30.1 kb and 18.6 kb on average, respectively. The number of genes present within the AT-blocks was also steady, and ranged between 233 and 236. In contrast, there was a drastic modification in genome architecture and genome size between *L. biglobosa* B3.5, sequenced using only Illumina, and G12-14 sequenced here. The two assemblies differed by more than 10% of their genome content. This difference corresponded to 3 Mb missing in B3.5 and recovered in G12-14 thanks to the nanopore long reads (Table 6). The difference mostly comprised of AT-blocks similar to those found in *L. maculans* and amounting to 15.5% of the G12-14 assembly vs. only 4.9% in the B3.5 assembly (Table 6). The increase in AT-block content is consistent with the lowering of the average GC content of the *L. biglobosa* genome in the new assembly (Table 4).

### Assessment of gene prediction

First, protein sequences of each genome (*L. maculans* JN3, *L. maculans* Nz-T4 and *L. biglobosa* G12-14) were obtained by translating gene CDS and aligned to protein databases (uniprot_sprot and uniprot_trembl 2016_11 release however *L. maculans* matches were excluded to prevent the introduction of known biases from the previous annotation effort) using blastp. Gene models for which proteins aligned with an e-value < 10^−10^ or with a CDS size > = 200 nt were retained.

Moreover gene completion for the newly generated annotations was evaluated using BUSCO^30^ v1.1b1 fungal and eukaryotic conserved genes databases. The relevance in the increase in gene number between the “old” and “new” gene prediction reflects a higher accuracy of the “new” annotation as evidenced by careful examination of genes encoding effectors. Effector genes encode for small (less than 300 amino-acids) secreted proteins (SSPs). They are usually poorly predicted *de novo* and may lack transcriptomic support due to expression in certain stages of parasitism only. The data obtained here drastically increased the number of predicted SSPs in both species (1,082 for *L. maculans* vs. 651 in the “old” annotation, 875 for *L. biglobosa* vs. 665 in the previous annotation) and drastically improved the gene models (data not shown). Also this new annotation allowed the identification of 75 genes hosted within the AT-block of the genome of *L. biglobosa*, previously unidentified (Table 6).

## Additional information

**How to cite this article**: Dutreux, F. *et al.* *De novo* assembly and annotation of three *Leptosphaeria* genomes using Oxford Nanopore MinION sequencing. *Sci. Data*. 5:180235 doi: 10.1038/sdata.2018.235 (2018).

**Publisher’s note**: Springer Nature remains neutral with regard to jurisdictional claims in published maps and institutional affiliations.

## Supplementary Material



## Figures and Tables

**Table 1:** Metrics of raw nanopore datasets.

		***Leptosphaeria maculans*** **JN3**	***Leptosphaeria maculans*** **Nz-T4**	***Leptosphaeria biglobosa*** **G12-14**
All	Number of reads	1,736,075 (23 flowcells)	883,625 (30 flowcells)	931,376 (19 flowcells)
	Cumulative size	6,044,436,091	4,813,632,244	3,362,956,224
	Estimated coverage	134X	107X	96X
	Average Size (bp)	3,482	5,448	3,611
	Longest read (bp)	1,612,597	1,769,119	949,732
	N50 (bp)	6,441	7,197	7,115
	# reads > 10Kb	72,482	76,176	61,856
1D	Number of reads	1,278,253	443,063	482,374
	Cumulative size	4,402,377,873	2,272,788,773	1,693,737,429
	Estimated coverage	98X	51X	48X
	Average Size (bp)	3,444	5,130	3,511
	Longest read (bp)	1,612,597	1,769,119	949,732
	N50 (bp)	6,431	7,497	7,250
	# reads > 10Kb	52,999	42,132	33,148
2D	Number of reads	457,822	440,562	449,002
	Cumulative size	1,642,058,218	2,540,843,471	1,669,218,795
	Estimated coverage	36X	56X	48X
	Average Size (bp)	3,587	5,767	3,718
	Longest read (bp)	237,404	301,948	84,061
	N50 (bp)	6,469	7,008	7,005
	# reads > 10Kb	19,483	34,044	28,708

**Table 2:** Metrics of illumina datasets.

	***Leptosphaeria maculans*** **JN3**	***Leptosphaeria maculans*** **Nz-T4**	***Leptosphaeria biglobosa*** **G12-14**
Number of reads	36,421,608	19,106,872	22,108,636
Cumulative size	8,089,956,155	4,399,409,082	4,198,089,452
Estimated coverage	180 X	98 X	120 X
Size (bp)	2×251 # HiSeq2500	2×251 # MiSeq	2×251# MiSeq

**Table 3:** Metrics of nanopore datasets used for genome assemblies.

status	***Leptosphaeria maculans*** **JN3**	***Leptosphaeria maculans*** **Nz-T4**	***Leptosphaeria biglobosa*** **G12-14**
	complete dataset	2D dataset	2D dataset
Number of reads	1,736,075	440,562	449,002
Cumulative size	6,044,436,091	2,540,843,471	1,669,218,795
Estimated coverage	134X	56X	48X
Average Size (bp)	3,482	5,767	3,718
Longest read (bp)	1,612,597	301,948	84,061
N50 (bp)	6,441	7,008	7,005
# reads > 10Kb	72,482	34,044	28,708

**Table 4:** Metrics of the existing and new assemblies.

Reference	***Leptosphaeria maculans*** **JN3**	***Leptosphaeria maculans*** **JN3**	***Leptosphaeria maculans*** **Nz-T4**	***Leptosphaeria biglobosa*** **B3.5**	***Leptosphaeria biglobosa*** **G12-14**
	Rouxel *et al.*^1^	This study	This study	Grandaubert *et al.*^2^	This study
# sequences	41	33	288	606	156
Cumulative size	44,892,605	45,986,477	43,426,637	31,788,051	34,950,111
N50	1,769,547	2,437,616	383,462	779,070	462,395
N90	1,020,521	1,391,278	64,152	125,916	123,107
L50	10	8	38	14	22
L90	22	18	150	49	77
# of N’s	1,128,152 (2.51%)	489,445 (1.06%)	0 (0%)	2,343,201 (7.37%)	0 (0%)
% GC	45.24%	45.22%	45.69%	51.39%	49.13%

**Table 5:** Metrics of the existing and new gene predictions.

Reference	***Leptosphaeria maculans*** **JN3**	***Leptosphaeria maculans*** **JN3**	***Leptosphaeria maculans*** **Nz-T4**	***Leptosphaeria biglobosa*** **B3.5**	***Leptosphaeria biglobosa*** **G12-14**
	Rouxel *et al.*^1^	This study	This study	Grandaubert *et al.*^2^	This study
# genes	12,611	13,047	14,026	11,390	12,678
# mono-exonic genes	2,931	5,204	5,898	2,726	4,630
Gene length (avg:med)	1,592:1,278	1,652:1,341	1,507:1,211	1,501:1,217	1,679:1,337
# exons per gene (avg:med)	2.94:2	2.28:2	2.20:2	2.67:2	2.35:2
CDS length (avg:med)	1,392:1,110	1,177:891	1,107:795	1,307:1,065	1,208:954
# introns	24,475	16,700	16,887	18,998	17,076
introns length (avg:med)	103:63	127:57	98:57	116:57	151:56
Coding fraction	38.9%	33.4%	35.8%	46.8%	43.8%
BUSCO (euk)	89%	98%	96%	93%	96%
BUSCO (fungi)	84%	96%	93%	90%	95%

**Table 6:** Genome compartmentalization of old and new assemblies.

Reference	***Lm*****JN3**^∗^	***Lm*****JN3**	***Lm*****Nz-T4**	***Lb*****B3.5**	***Lb*****G12-14**	
Rouxel *et al.*^1^	This study	This study	Grandaubert *et al.*^2^	This study		
GC blocks	# blocks	399	534	587	389	318
	% assembly	64	63.1	66.6	95.1	84.5
	mean size (kb)	70.4	54.4	49.2	77.7	92.9
	stdev (kb)	—	63.1	61.9	226	157
	min (kb)	1	1	1	1	1
	max (kb)	500	431	349	1708	1180
	# genes	—	12,892	13,790	11,361	12,603
AT blocks	# blocks	413	564	779	324	335
	% assembly	36	36.9	33.4	4.87	15.5
	mean size (kb)	38.6	30.1	18.6	4.8	16.2
	stdev (kb)	—	48.1	23.5	10.7	17.7
	min (kb)	1	1	1	1	1
	max (kb)	320	319	245	120	142
	# genes	—	233	236	29	75
^∗^Results from Grandaubert *et al.* AT rich regions were manually curated.						
